# Multisensory and Motor Representations in Rat Oral Somatosensory Cortex

**DOI:** 10.1038/s41598-018-31710-0

**Published:** 2018-09-10

**Authors:** Ann M. Clemens, Yohami Fernandez Delgado, Max L. Mehlman, Poonam Mishra, Michael Brecht

**Affiliations:** 1 Neural Systems & Behavior, Marine Biological Laboratory, 7 MBL Street, Woods Hole, MA 02543 USA; 2 Bernstein Center for Computational Neuroscience Berlin, Humboldt-Universität zu Berlin, Philippstr. 13, Haus 6, 10115 Berlin, Germany; 3 Department of Biology, Wake Forest University, Winston-Salem, NC 27106 USA; 4 Department of Psychological and Brain Sciences, Dartmouth College, Hanover, NH 03755 USA; 5 Cellular Neurophysiology Laboratory, Molecular Biophysics Unit, Indian Institute of Science, Bangalore, 560012 India

## Abstract

In mammals, a complex array of oral sensors assess the taste, temperature and haptic properties of food. Although the representation of taste has been extensively studied in the gustatory cortex, it is unclear how the somatosensory cortex encodes information about the properties of oral stimuli. Moreover, it is poorly understood how different oral sensory modalities are integrated and how sensory responses are translated into oral motor actions. To investigate whether oral somatosensory cortex processes food-related sensations and movements, we performed *in vivo* whole-cell recordings and motor mapping experiments in rats. Neurons in oral somatosensory cortex showed robust post-synaptic and sparse action potential responses to air puffs. Membrane potential showed that cold water evoked larger responses than room temperature or hot water. Most neurons showed no clear tuning of responses to bitter, sweet and neutral gustatory stimuli. Finally, motor mapping experiments with histological verification revealed an initiation of movements related to food consumption behavior, such as jaw opening and tongue protrusions. We conclude that somatosensory cortex: (i) provides a representation of the temperature of oral stimuli, (ii) does not systematically encode taste information and (iii) influences orofacial movements related to food consummatory behavior.

## Introduction

Everybody knows a warm coke makes a horrible drink. In neural terms, however, we do not understand why this is the case. The reason for our ignorance about multisensory stimulus properties is related to the fact that the vast majority of studies in sensory neuroscience focus on single modalities. As studies emerge that consider multisensory and multimodal interactions, an interesting, albeit complex, picture emerges; this is especially true for the case of oral stimuli.

The analysis of gustatory representations in rodent cortex has seen dramatic advances in recent years. Early work identified gustatory responses in the gustatory cortex^1–3^. Subsequent imaging work in rats provided the first evidence for and spatial segregation of responses to different tastes in gustatory cortex although no region was found to be specific to a single modality. These gustatory responses were observed lateral from mechanosensitive responses to tongue stimulation^4^. Later work in mice extended these findings and provided compelling evidence for a strongly gustato-topic organization of mouse gustatory cortex, where spatially distinct cortical sites respond to sweet, bitter and salty stimuli^5^. Importantly, these authors also showed that the mere stimulation of these cortical neurons suffices to evoke the appropriate behavioral responses to the respective gustatory stimuli^6^. Other investigations, however, have challenged the degree of spatial segregation of gustatory taste representations^7^. A separate line of work has demonstrated that gustatory cortex neurons can display taste-specific responses that vary as a function of time since stimulus delivery^8^ and that taste-specific information can be encoded by the coordinated activity of neuron pairs^9^. The gustatory cortex can also encode non-gustatory stimuli like tactile, thermal and olfactory information during the consumption of food^10–12^. Samuelsen and Fontanini (2017) demonstrated that these cortical neurons can integrate chemosensory stimuli by responding exclusively to tastants and odorants or the combination of both^13^. These findings suggest that the gustatory cortex encodes information in a dynamic, distributed and multimodal manner^12–14^.

The representation of non-gustatory properties of oral stimuli is also complex and not well understood. Work in primates suggested that, at least in orbitofrontal cortex, neural responses are highly tuned to the texture and mechanical properties of food^15^. Early multisensory work in rodents suggested a segregation of mechanically-driven, temperature-selective and gustatory responses^16,17^. Specifically, these authors suggested that mechanically-driven responses are found in medial granular cortex (putatively medial somatosensory cortex), while temperature-selective responses locate to lateral granular cortex and gustatory responses locate to agranular cortex^16^. Such a segregated processing scheme immediately raises the question, how and where are different modalities integrated?

Food stimuli can evoke expressive facial movements of pleasure and disgust^18–21^. Neural control of such facial expressions is likely mediated by subcortical pattern generators^22,23^, but potential cortical contributions to these orally-evoked movements are largely unknown. Tracing and stimulation work identified cortical regions involved in tongue movements^24^, but these appear to be separate from gustatory cortex and oral somatosensory cortex.

In our study, we investigated unresolved issues regarding multisensory integration of stimuli in oral somatosensory cortex. We performed *in vivo* whole-cell recording and motor mapping experiments in oral somatosensory cortex to address the following questions: (i) What are the tactile response properties in oral somatosensory cortex? (ii) Do neurons in oral somatosensory cortex also respond to temperature and taste stimuli? (iii) Does oral somatosensory cortex contribute to orofacial movements?

## Materials and Methods

All experiments complied with German and American regulations on animal welfare and were approved by the Landesamt für Gesundheit und Soziales in Berlin, Germany and the Institutional Animal Care and Use Committee in Woods Hole, USA respectively.

### Animal Preparation

#### Whole-cell recording experiments

Long-Evans male rats (P21–P28, n = 39) were anaesthetized using urethane (1.4 g/kg i.p.). Animals were confirmed to be fully anaesthetized when there was no response to pinching of the paw or tail. Subsequent to full anaesthesia, the head was secured with stereotaxic ear bars. Incised tissue was locally anaesthetized with lidocaine. A rectal probe monitored body temperature, and a homeothermic blanket (FHC, Bowdoinham, Me., USA) maintained it at 37 ± 0.5 °C. A craniotomy was made above the oral somatosensory cortex (3–4 mm anterior to bregma; 6–7.5 mm lateral to bregma). Electrodes entered oral somatosensory cortex from above at a ca. 30° angle from the axis perpendicular to the cortical surface. Recording sites were verified by removing the patch electrode after the recording and re-inserting a tungsten electrode to the location and depth of the whole cell recording. A lesion was made and recording coordinates were confirmed by examination of recording sites in flattened cytochrome oxidase stained sections.

#### Stimulus Delivery System

**Figure 1:**
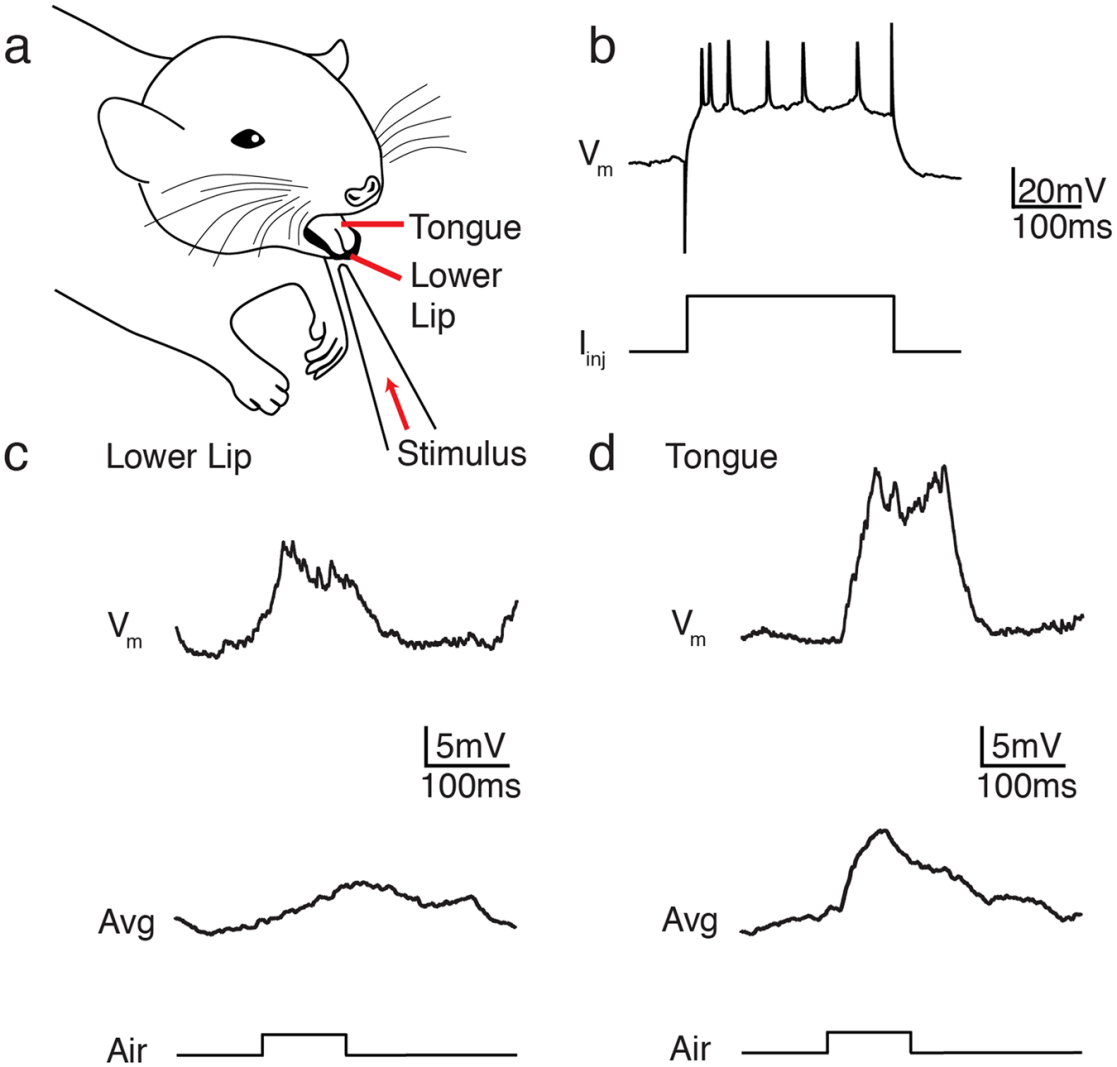
Stimulation of intra-oral receptive fields in somatosensory cortex (S1). **(a**) Stimulated area of an anaesthetized rat. The lower lip or tongue was stimulated with an air puff generated by a computer-triggered airflow controller. **(b)** Representative voltage trace (V_m_) from a whole-cell patch clamp recording from an S1 cortical neuron during current injection (I_inj_). **(c,d**) Example whole-cell recordings from a representative S1 cortical neuron showing a single trial response (*top*) and averaged response (*bottom*; n = 19 trials) to air puff stimulation of the lower lip (**c**) or tongue (**d**). Timing of stimulus delivery is indicated below each trace.

In order to establish the oral sensory receptive field, puffs of air were presented to various peri- and intra-oral regions; these stimuli lasted 0.1 s and were separated by a 5 s interstimulus interval (Fig. 1c,d). Air puffs were generated from pulses of compressed air, delivered by a computer-triggered airflow controller (Sigmann Electronics, Germany). Air puffs, as well as the liquid stimuli described below, were delivered via a stiff micropipettor tip with a 1 mm opening positioned 5–10 mm rostrolateral from the mouth and pointing to the lower lip or tongue area (Fig. 1a).

#### Adjustment of thermal and gustatory stimuli

Gustatory and thermal stimuli consisted of 100 μl of liquid rapidly ejected onto the animal’s tongue. Liquids were loaded into the same micropipettor tips used for the above described air puff stimuli and were ejected by the same 0.1 second-long pulses of compressed air.

Gustatory stimuli consisted of 100 mM sucrose solution as a sweet stimulus, 0.5 mM quinine solution as a bitter stimulus^6^, and tap water as a neutral stimulus. In separate behavioral experiments, we offered the sucrose and quinine solutions to four rats, littermates to our experimental animals. We found these animals to be very keen to lick this sucrose solution, a behavioral pattern suggesting that the sucrose solution had rewarding value. Subsequently, we offered quinine solution and after 2 to 3 lick bouts the animals refused to lick further, shook their heads and wiped their tongues with their paws; this behavioral pattern suggests that quinine solution had aversive taste characteristics.

Cold stimuli ranged from 3–4 °C, room temperature stimuli from 20–21 °C and hot stimuli 85–90 °C. We used room temperature water as a neutral stimulus; cold stimuli were loaded from a syringe filled with ice-cold water stored on crushed ice; hot stimuli were loaded from a syringe filled with very hot freshly microwaved (i.e. close to boiling) water. Cold, room temperature and hot stimuli were ejected directly after loading. In psychophysical experiments we found that such stimuli squirted on the back of the hand of human observers were difficult to discriminate when we applied ≤50 μl liquid pulses. The low thermal discriminability of such quantities might be related both to the low absolute volume and the rapid equalization of the liquid’s temperature during the application process. When we applied 100 μl liquid pulses human subjects described the resulting sensations as cold, neutral and hot. Only contacts with much larger liquid quantities were described as pleasantly cold or painfully hot. Accordingly, we consistently applied 100 μl liquid pulses as cold, neutral and hot stimuli in our experiments.

### *In vivo* whole-cell blind patch recordings

We used standard physiological techniques previously described for whole-cell recordings^25,26^. Pipettes were pulled to 3–8 MΩ (P1000, Sutter Instruments, Novato, Calif., USA) from filamented (0.25 mm) borosilicate glass (OD 2.0 mm, ID 1.5 mm, Hilgenberg, Malsfeld Germany). Intracellular solution was composed of (in mM) K-gluconate 130, Na-gluconate 10, HEPES 10, phosphocreatine 10, MgATP 4, GTP 0.3, NaCl 4 and biocytin 0.3–1% at pH 7.2. Signals were amplified (Cornerstone-amplifier, Dagan Corporation, Minneapolis MN USA), filtered at 3–10 kHz and digitized at 20 kHz (ITC-16; Instrutech, New York, N.Y., USA) using HEKA (Lambrecht, Germany) software. Recorded membrane potential traces in response to different stimuli types were exported and analyzed in Igor Pro (Wavemetrics Inc., Portland, OR, USA).

### Analysis

For each cell, the post-synaptic potential (PSP) amplitude associated with each stimulus condition (i.e. air puff; gustatory and thermal liquid stimuli) was calculated from the average trace of multiple trials. Excitatory post-synaptic potential (EPSP) amplitude was quantified as the difference between the peak depolarization observed in response to the stimulus and the cell’s resting membrane potential. Rise time to half peak was used to quantify response kinetics, calculated as the time duration required to reach 50% of the EPSP amplitude. 20–80% rise time was determined by measuring the duration of time between 20% and 80% of the maximum EPSP amplitude.

#### Motor mapping experiments

Long-Evans male rats (P28-P35, n = 6) were initially anaesthetized using ketamine (90 mg/kg i.p.) and xylazine (10 mg/kg i.p). After the initial anaesthesia, we supplemented ketamine (5% of the initial dose, as needed) and acepromazine (0.2 mg/kg i.p). For motor mapping, a larger craniotomy ranging from 0.5–5.5 mm anterior to bregma and 4–10 mm lateral to bregma was made. Tungsten microstimulation electrodes entered cortex from above at a ca. 30° angle from the axis perpendicular to the cortical surface and were lowered to a depth of 1.5 mm. In each experiment, stimulation was delivered to 16 or 40 sites arranged in a grid with a spacing of 0.5 or 1 mm. The stimuli consisted of a train of 60 pulses, each 0.3 ms in duration applied at a frequency of 200 Hz; this resulted in a 300 ms long stimulation train which was delivered repeatedly with an interstimulus interval of 5 s. Unipolar pulses (electrode tip negative) were applied at current intensities ranging from 10 μA to 250 μA. Similar stimulation current ranges were used previously to elicit motor responses by activation of the somatosensory cortex^27,28^. The stimulation intensity was initially set low (10 μA) then gradually increased until a motor response was observed; this procedure revealed the response threshold for each site. Once the response threshold was determined, repeated trains of stimulation at this intensity were delivered to verify that motor responses occurred repeatedly, consistently and in a manner time-locked to stimulation onset. If the stimulation intensity reached 250 μA without eliciting a motor response, the site was designated as “no response”.

### Histochemical visualization of barrels and other granular modules in somatosensory cortex

Animals were deeply anaesthetized with an additional dose of urethane (1.4 g/kg i.p.) and perfused transcardially with prefix, followed by 4% paraformaldehyde (PFA). Brains were removed, hemispheres were separated and cortices were flattened between two glass slides separated by clay spacers. Glass slides were weighed down with small ceramic weights for ca. 3 hr. Afterwards, flattened cortices were stored overnight in 2% PFA and 80 μm tangential sections were cut on a vibratome. Sections were stained for cytochrome-oxidase reactivity using the protocol of Wong-Riley (1979)^29^. The cytochrome-oxidase technique was used to identify the granular somatosensory cortex in combination with electrolytic lesions and reconstruction of electrode penetrations. These techniques allowed us to assign microstimulation effects unequivocally to the orofacial somatosensory cortex.

## Results

### Identification of intra-oral receptive fields in somatosensory cortex

Intra-oral receptive fields of primary somatosensory cortical neurons were determined by applying tactile stimulation (i.e. air puffs) to different locations and recording the elicited membrane potential responses. Figure 1 illustrates the voltage response of one cortical neuron to air puff stimuli targeting the lower lip or the center of the tongue. Current injection (Fig. 1b) identified the cell as a regular spiking neuron. Post-synaptic potentials elicited by air puff stimuli are shown below (Fig. 1c,d). Tongue stimulation produced higher amplitude and more rapid responses compared to lower lip stimulation (Fig. 1c,d, single trial, *top*). This difference was confirmed by averaging the response of 19 trials in each condition (Fig. 1c,d, averaged response, *bottom;* Tongue: response amplitude = 10.3 mV, time to peak = 7.0 ms; Lower Lip: response amplitude = 4.8 mV, time to peak = 11.6 ms). Thus, this neuron showed a strong response preference for tongue stimulation over lip stimulation, even though lips and tongue are only separated by a few millimeters on the body.

### Temperature- and taste-dependent responses of oral somatosensory cortex

**Figure 2:**
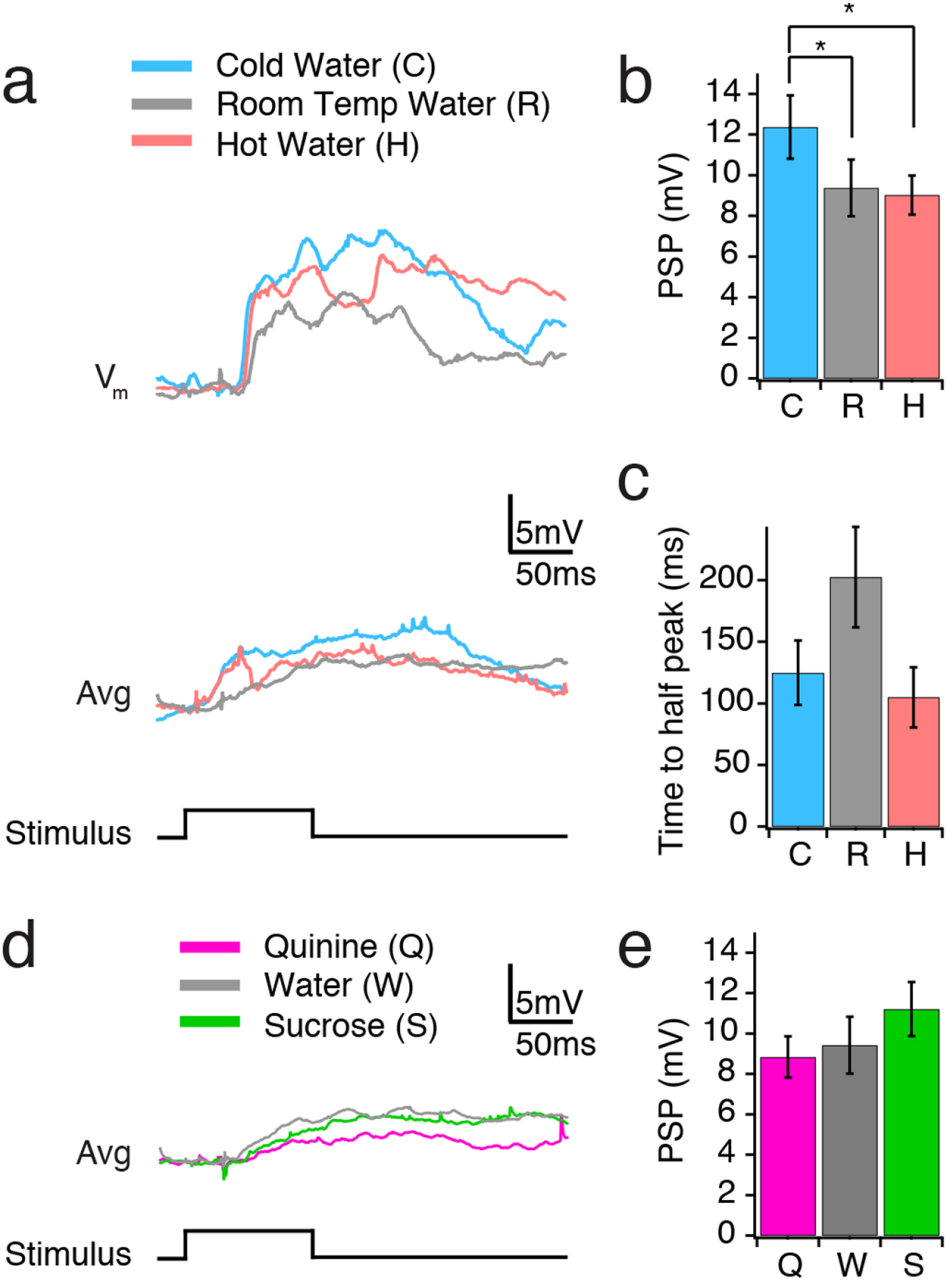
Temperature- and taste-dependent responses of oral somatosensory cortex. **(a)** Average membrane potential responses to cold (C), room temperature (R) and hot (H) water of a single cell (averaged over multiple) (*top*) and all cells (averaged over multiple trials across all cells) (*bottom*; cold and room temperature: n = 20 cells; hot: n = 19 cells). Timing of water stimulus is indicated below. **(b)** Mean post-synaptic potential (PSP) amplitude for all cells in response to cold (C), room temperature (R) and hot (H) water. **(c)** Mean time to half peak in response to cold (C), room temperature (R) and hot (H) water. **(d)** Average membrane potential responses to quinine (Q), water (W) and sucrose (S) for all cells (n = 21 cells). Timing of liquid stimulus is indicated below. **(e)** Mean PSP amplitude for all cells in responses to quinine (Q), water (W), and sucrose (S). Bar graphs are mean ± SEM; *p < 0.05.

In order to address whether multisensory information is encoded in the oral somatosensory cortex, we recorded membrane potential responses to liquids with distinct temperatures and tastes applied to the animal’s tongue. Before liquid stimuli were applied, responses of all neurons were first tested with air puff stimuli to ensure that they responded to tactile stimulation. When water stimuli were applied, we found that neurons within the oral somatosensory cortex respond differentially to temperature, where responses to cold stimuli were largest (Fig. 2a,b; cold: n = 20 cells, 12.5 ± 1.6 mV; room temperature: n = 20 cells, 9.4 ± 1.4 mV; hot: n = 19 cells, 9.1 ± 1.0 mV; repeated measures ANOVA p = 0.015; pairwise multiple comparison Holm-Sidak method: cold vs. hot p = 0.015, cold vs. room p = 0.027, hot vs. room temperature p = 0.659). There was no difference in response kinetics across the different temperature stimuli. We confirmed this with the measurement of time to half peak EPSP amplitude (Fig. 2c; cold: n = 20 cells, 125 ± 26 ms; room temperature: n = 20 cells, 203 ± 41 ms; hot: n = 19 cells, 106 ± 24 ms; Friedman repeated measures ANOVA on ranks p = 0.104) as well as measurement of the 20–80% rise time (cold: n = 20 cells, 113 ± 33 ms; room temperature: n = 20 cells, 190 ± 52 ms; hot: n = 19 cells 117 ± 33 ms; One-Way Repeated Measures ANOVA p = 0.128).

We additionally asked whether taste information might be represented in oral somatosensory neurons. We compared post-synaptic responses to bitter (quinine 0.5 mM), water and sweet (sucrose 100 mM) stimuli. Overall, we saw little difference in neuronal responses to taste. There was no difference in response amplitude (Fig. 2d,e, left; quinine: n = 21 cells, 8.8 ± 1.0 mV; water: n = 21 cells 9.1 ± 1.4 mV; sucrose: n = 21 cells 11.2 ± 1.3 mV; repeated measures ANOVA p = 0.373). There was a difference in response kinetics for sucrose compared with water stimuli, but this difference was not apparent in the averaged traces (Fig. 2d; quinine: n = 21 cells, 127 ± 43 ms; water: n = 21 cells, 215 ± 39 ms; sucrose: n = 21 cells, 136 ± 39 ms; Friedman repeated measures ANOVA on ranks p = 0.041; Wilcoxon Signed Rank Test: quinine vs. sucrose p = 0.898, quinine vs. water p = 0.083, sucrose vs. water p = 0.037). Hence, we conclude that oral somatosensory cortex is not robustly sensitive to sweet or bitter taste stimuli, though future studies examining the suprathreshold impact of small differences in subthreshold temporal responses should be performed.

### Stimulation of oral somatosensory cortex elicits movements related to food consummatory behavior

**Figure 3:**
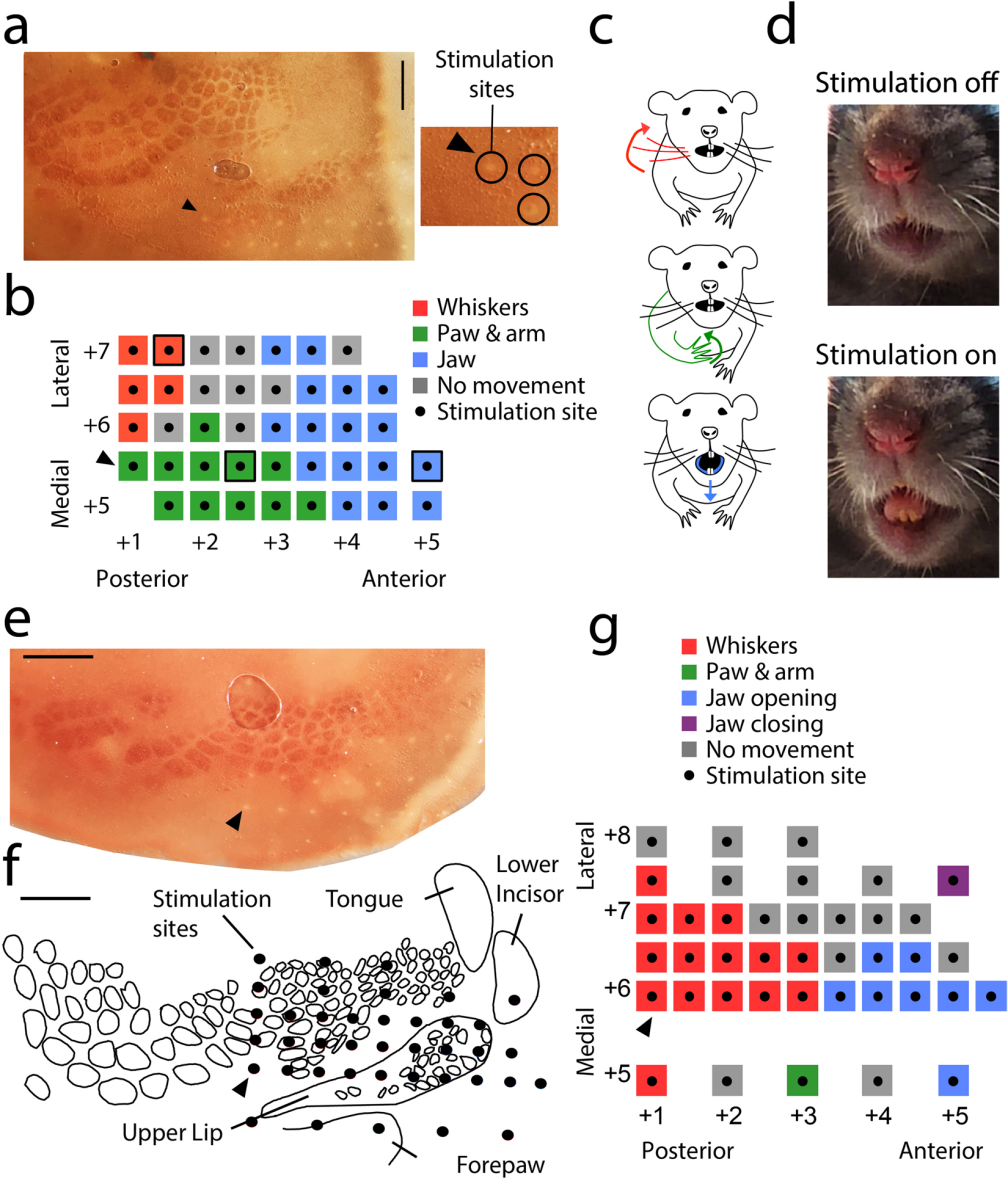
Stimulation of oral somatosensory cortex elicits movements related to food consummatory behavior. **(a)** Photomicrograph of a tangential section of flattened somatosensory cortex stained for cytochrome-oxidase reactivity. Stimulation sites appear as small white dots in the tissue. The alignment between this photomicrograph and the map in *b* is indicated by a black arrowhead in each panel pointing to corresponding locations. Examples of visible stimulation tracks are magnified and circled. Scale bar: 1 mm. **(b)** Map of motor responses evoked by electrical stimulation of oral somatosensory cortex and surrounding regions. Numbers on the bottom and the left indicate the anterior/posterior and medial/lateral location of stimulation sites, respectively, in mm relative to bregma. The color surrounding each stimulation site indicates the associated motor response. **(c)** Schematic diagrams of motor responses displayed by the whiskers (*top*), paw and arm (*middle*), and lower jaw (*bottom*). The stimulation sites producing these responses are indicated by the black outlines in *b*. **(d)** Photographs of the animal’s mouth when stimulation is off (*top*) or on (*bottom*). The stimulation site producing this response is indicated by the black outline in *b*. Also, see Supplementary Video 1 for a representative orofacial movement pattern evoked by stimulation of somatosensory cortex. **(e)** Photomicrograph of a tangential section of flattened somatosensory cortex stained for cytochrome-oxidase activity from a second motor mapping experiment. Stimulation sites appear as small white dots in the tissue. The alignment between this photomicrograph and the map in f and g is indicated by a black arrowhead in each panel pointing to corresponding locations. Scale bar: 1 mm. **(f)** Reconstruction of the somatosensory map from a series of tangential sections. Stimulation sites are indicated as black dots. Scale bar: 1 mm. **(g)** Map of motor responses evoked by electrical stimulation of oral somatosensory cortex and surrounding regions. Numbers on the bottom and the left indicate the anterior/posterior and medial/lateral location of stimulation sites, respectively, in mm relative to bregma. The color surrounding each stimulation site indicates the associated motor response.

In order to study the control of orofacial movements we applied microstimulation to the left oral somatosensory cortex (Fig. 3a). In the experiment shown we stimulated 40 sites spanning from 1–5 mm anterior to bregma and from 5–7 mm lateral to bregma; 32 (80%) stimulation sites evoked a motor response (Fig. 3a,b). Motor responses to stimulation were assessed at the time of the experiment and recorded with video for post-hoc analysis. Areas neighboring the oral somatosensory cortex, including the paw and whisker regions, were stimulated in order to provide orientation with respect to the target oral region. The most posterior and lateral sites evoked whisker responses when stimulated with an intensity of 200–250 μA (Fig. 3b, red); these responses consisted of a backward “twitch-like” movement of the right whisker rows C, D and E (Fig. 3c, *top*). Posterior sites more medial elicited motor responses in the paw and arm with a threshold of 100–150 μA (Fig. 3b, green). These responses predominately consisted of an upward movement of the right paw and arm, with the paw rotating inward (medially) at the wrist (Fig. 3c, *middle*); however, a minority of stimulation sites produced different movements in which the paw and arm rotated outward (laterally) or moved forward and downward; these different responses did not appear to be topographically organized. All of these responses occurred at the time of stimulation onset, with the paw and arm returning to their resting position 1–2 sec after stimulation offset. Finally, anterior stimulation sites evoked downward jaw movements causing the mouth to open upon stimulation onset (Fig. 3b, blue; 3c, *bottom*; 3d); 1–2 sec after stimulation offset, the mouth returned to its relatively closed resting position. Among these sites eliciting jaw movements, the medial sites generally had a lower threshold (50–150 μA) compared to more lateral sites (200–250 μA). A representative example of the observed mouth opening movements is provided (Supplementary Video 1). In a second representative stimulation experiment (Fig. 3e–g), we reconstructed the somatotopic map from a series of tangential sections and superimposed the visible stimulation sites (Fig. 3f). We confirmed that stimulation sites which evoked jaw movements coincided with oral regions of the somatosensory map (Fig. 3g). In this experiment, one lateral stimulation site close to gustatory cortex produced an upward movement of the jaw in response to 200 μA of stimulation, causing the mouth to fully close upon stimulation onset then return to its resting position 1–2 sec after stimulation offset. All responses observed in this experiment were elicited repeatedly, consistently, and in a manner time-locked to the onset of stimulation.

Four further experiments led to similar conclusions. On average, we observed motor responses in 70.5 ± 11% of stimulation sites. The most posterior and lateral sites elicited backward movement of the right whisker rows D and E. Medial stimulation sites evoked motor responses in the paw with a threshold of 100–150 μA; these responses consisted of an upward movement of the right digits. Finally, the most anterior sites elicited a downward jaw movement when stimulated with an intensity of 100–200 μA; the mouth opened upon stimulation onset then returned to its relatively closed resting position 1–2 sec after stimulation offset. In general, the most lateral stimulation sites (7–8 mm lateral to bregma) failed to evoke motor responses potentially because the threshold for movement initiation was increased. In two experiments, the lower jaw was held open to observe movements of the tongue which we filmed with high-speed videography. Here, we saw overt protrusions of the tongue with stimulation in regions which overlapped with jaw movements. This movement of the tongue resembles licking behaviors seen in awake animals.

Together, our experiments provide a motor map of the oral somatosensory cortex and surrounding regions. Importantly, the topography of motor responses evoked by cortical stimulation of these regions generally corresponds to the topography of their receptive fields determined from cortical recordings^30,31^. Additionally, we used histological techniques (cytochrome oxidase reactivity stains with electrolytic lesions and visible electrode penetrations) to assign the observed stimulation effects to oral somatosensory cortex. Thus, the regions of somatosensory cortex with receptive fields in the paw, whiskers or mouth evoke motor responses in the paw, whiskers or mouth, respectively, when electrically stimulated. In the current study, neurons responsive to various stimuli applied to the mouth were recorded in the region of somatosensory cortex that produced mouth opening and tongue movements when electrically stimulated. Significantly, we show that oral somatosensory cortex encodes food-relevant temperature information and that stimulation of these sensory encoding regions produces pro-consummatory motor behaviors.

## Discussion

In this study, we identified receptive fields of oral somatosensory cortex and found that: (i) neurons in oral somatosensory cortex show multisensory responses to stimuli of varying temperature, with the largest post-synaptic responses associated with cold liquid stimuli, (ii) neurons do not systematically encode taste information and (iii) orofacial motor movements resembling food consumption behavior are elicited by microstimulation of oral somatosensory cortex.

Using *in vivo* whole-cell intracellular recording techniques, we confirmed the specificity of receptive fields of the intra-oral somatosensory cortex. Movement of the air puff stimulus by just a few millimeters from the tongue to lower lip region dramatically reduced the post-synaptic response (Fig. 1). Such a “sharp” receptive field compares to what is observed in layer 4 of barrel cortex^26^, where movement of stimulation by a few millimeters greatly reduces sub-threshold responses. This suggests that the oral somatosensory cortex is highly tuned for active sensation of small features of oro-tactile stimuli, which a rat is likely to encounter during natural exploration. Much like the regional preferences of the tongue in terms of different threshold sensitivity to gustatory stimuli, or different taste modalities^32–34^, the observed small receptive fields for tactile stimuli may provide for further specialization of tongue regions for sensing the tactile properties of food as well as facilitate integration of other relevant food-related features such as taste and temperature.

Although air puff stimuli provide for easy and precise mapping of somatotopic regions, it is more likely that sensation of the mouth region is tuned to things more palatable. Temperature, among many other food modalities (e.g. texture and olfactory properties) could significantly influence the palatability of food^35–39^. Thus, using the aforementioned method of liquid delivery, we found that the amplitude of post-synaptic responses varied significantly with temperature. In the trigeminal ganglion, temperature responses are segregated across different types of thermosensory neurons, with some neurons tuned to hot stimuli and others tuned to cold stimuli^40^; in contrast, we found that oral somatosensory cortex neurons consistently displayed a larger average amplitude of post-synaptic subthreshold depolarization in response to cold water compared with room temperature or hot water. It is possible that the responses to water delivery could be partially elicited by the tactile component of the stimulation. If these responses were entirely driven by tactile stimulation, then we would expect similar responses to the three types of water stimuli, which all share the same tactile properties. However, we observed differential responding, with heightened responses to cold water compared with room temperature or hot water, suggesting that the neurons indeed encoded information pertaining to stimulus temperature. Our findings are in line with a previous report showing that layer 2/3 neurons in mouse forepaw somatosensory cortex are capable of responding to both cooling and tactile stimuli^40^ and another report that showed changes in neuronal activity in the somatosensory cortex with alteration of scrotal skin temperature^41^. The mechanism of the augmented post-synaptic response to cold-temperature liquids may occur through cool-temperature sensitive *TRPM8* channels^42,43^. In the trigeminal ganglion, thermosensory neurons responsive to cold stimuli express *TRPM8*^40^, and it is possible that output from these subcortical sensory neurons contributes to the cold water responses we observed in the oral somatosensory cortex. Indeed, in mice lacking *TRPM8*, neurons in the forepaw somatosensory cortex no longer respond to cooling stimuli^41^. In regards to multisensory integration, temperature processing has been shown to interact with taste processing at subcortical levels. Electrophysiological recordings from the rat geniculate ganglion showed that temperature modulates taste responsiveness and in some neurons, it is an activation stimulus^44^. Similar results were reported behaviorally suggesting that rats preferred sucrose at 20 °C, while cold temperatures reduced sucrose palatability^39^. The studies described above reveal important mechanisms of temperature processing at subcortical levels; however, the manner in which temperature is further processed and represented at cortical levels is relatively less clear. Our findings provide valuable insights into this issue, demonstrating that neurons in the oral somatosensory cortex display an amplified response to cold temperature compared with room or hot temperatures.

We also observed membrane potential responses to liquids of different tastes, though no robust differences were apparent. We observed a few oral somatosensory neurons that responded preferentially to sweet (sucrose) or bitter (quinine) stimuli, however, the result overall was no average difference in post-synaptic response amplitude. Future studies regarding possible taste sensitivities in oral somatosensory cortex may benefit from the examination of a wider variety of taste stimuli, such as NaCl which is transduced through amiloride-sensitive sodium channels located in the rat tongue^45^. Furthermore, it may be possible that more specialized regions of the rat oral cavity may exhibit taste sensitivity in the somatosensory cortex, such as the nasoincisor ducts or circumvallate papilla.

Lastly, we performed microstimulation experiments with histological verification to study the control of orofacial movements by the somatosensory cortex. Electrical stimulation has been extensively used to study the neural basis of behaviors, however, it is important to consider the methodological limitations of the microstimulation technique. This includes the direct activation of multiple neurons surrounding the electrode and that it is impossible to determine the number and type of stimulated neurons. It is also important to consider the amount of current applied and the stimulation parameters because the evocation of different behavioral responses depends on stimulus characteristics^46,47^. For a more detailed explanation of the methodological shortcomings see Tehovnik (1996). Stimulation protocols similar to ours have been used in the somatosensory cortex of rats to elicit motor responses^27,28^.

In the present study, we found that stimulation of oral somatosensory neurons produces small orofacial movements including jaw opening and tongue protrusions. Expression of similar facial patterns has been reported with stimulation of the caudate brainstem in decerebrate animals^48^. Rhythmic jaw movements have also been observed with stimulation of cortical masticatory A-area (orofacial motor cortex) and P-area (posterior area in the insular cortex). Stimulation also induced salivation in cortical masticatory P-area^49^. Additionally, jaw movements have been previously reported with stimulation in rostral regions of the primary somatosensory cortex^27,50,51^. The identified movement-initiating regions in our study appear to overlap with those previously identified by Uchino and collaborators (2015). These results corroborate previous findings suggesting that rostral activation of the primary somatosensory cortex has an important role in controlling jaw movements by stimulating direct descending projections to the premotor neurons. Thus, the elicitation of complex facial expressions involves an interaction between cortical regions and brainstem nuclei.

We found that regions of the somatosensory cortex responding to complex features of stimuli such as temperature are overlapping with those capable of initiating orofacial movements that resemble those seen during food consumption behavior. How the small motor movements we observed may fit together with the complex expressions made by awake animals is unclear. We speculate, however, that such movements may provide a direct link between the perception of haptic/temperature varying stimuli and the outward/facial motor expression of whether the stimuli are deemed favorable or not.

## Electronic supplementary material

Supplementary Video
